# Minimal improvements in the global burden of skin disease from 1990 to 2013

**DOI:** 10.1016/j.jaad.2016.08.006

**Published:** 2017-01

**Authors:** Jessica Mounessa, Taylor Braunberger, Cory A. Dunnick, Robert P. Dellavalle

**Affiliations:** a Department of Dermatology, University of Colorado Anschutz Medical Campus, Aurora, Colorado; b University of North Dakota School of Medicine, Fargo, North Dakota; c Department of Community and Behavioral Health, Colorado School of Public Health, University of Colorado Anschutz Medical Campus, Aurora, Colorado; d Dermatology Service, Eastern Colorado Health Care System, US Department of Veteran Affairs, Denver, Colorado

*To the Editor:* Skin disease often impairs quality of life and results in an increase in years lived with disability (YLDs). In 2010, skin disease was the fourth cause of nonfatal disease burden worldwide.^1^ Skin conditions ranked between the second and eleventh leading causes of YLDs globally.^1^ We investigated changes in the global burden of skin disease experienced in developed and developing countries between 1990 and 2013.

We used the Institute for Health Metrics and Evaluation (IHME) reports of Leading Causes of YLDs, 1990 and 2013.^2^ The percent change in skin diseases between 1990 and 2013 was collected for each country listed. Of the 187 reported countries, we identified 47 developed countries according to the 2015 Human Development Index.^3^ The greatest improvements were seen in Portugal, with a 9% decrease in disability between 1990 and 2013. Israel, Spain, the Czech Republic, and Hungary followed the lead with a 7% decrease in YLDs. In addition, 6 of the 47 developed countries did not see any changes in YLDs from skin conditions, and 11 experienced an increase in their YLDs (Fig 1). The mean percent change seen in developed countries was a 1.85% improvement in disability (standard deviation [SD] = 3.73 [95% confidence interval {CI}, −2.92 to −0.78]).

Of the 140 developing countries, 26 reported improvements in YLDs from skin diseases, as indicated by a negative percent change from 1990 to 2013 (Fig 2). The average percent change in developing countries was a 2.6% decrease in disability (SD = 3.75 [95% CI, 2.04-3.29]). Ten countries saw no change, and the majority of developing countries (n = 104) experienced an increase in disability. Nicaragua, Oman, and Palestine saw the greatest increase in percent change of disability (11%, 11%, and 15%, respectively).

Skin diseases continue to affect not only those living in developing countries, but also those living in developed countries. The decrease experienced in the United States was low (1%), especially when compared to other developed countries. In other words, the percentage of Americans living in less than ideal health because of skin diseases has not changed substantially in the past 23 years, even though the United States has been spending far more on health care than any other developed country.^4^

An increase in life expectancy of Americans may partially account for a larger number of people living in the United States with skin diseases. Yet most of the developed countries have also experienced an increase in life expectancy. This therefore does not fully explain why the United States lags behind the majority of developed countries in terms of improvements in disability from skin disease. In addition, Americans may have higher expectations regarding treatment of skin diseases as a result of direct-to-consumer pharmaceutical advertising, which may overemphasize potential benefits of medications.^5^ Patient expectations are unaccounted for in the IHME data.

Over the past 2 decades, the majority of both developed and developing countries have experienced little or no improvement in disability caused by skin diseases. Health care providers around the world must shift their focus to improving the quality of lives in their patients with skin diseases.

## Figures and Tables

**Fig 1:**
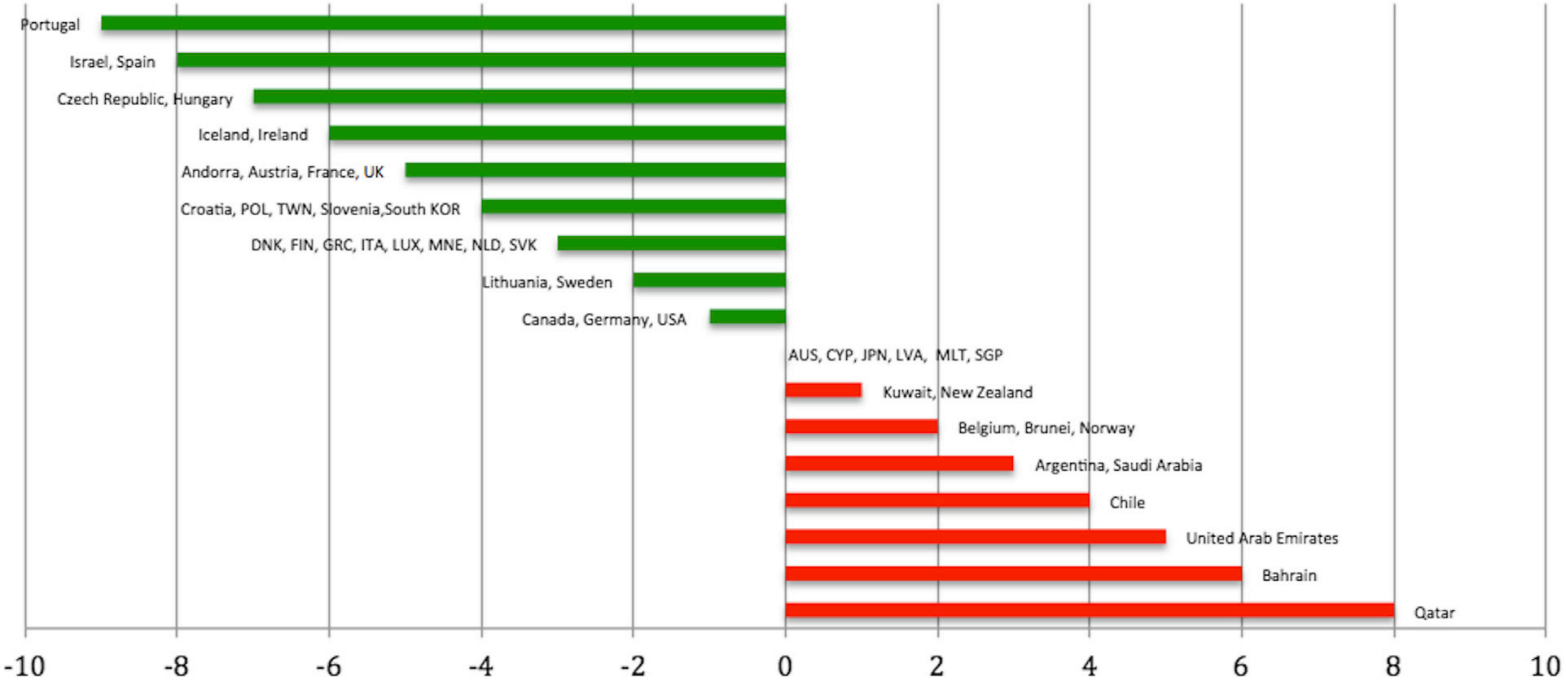
Percent change in disability-adjusted life years for top 10 and bottom 10 developed countries. Negative values indicate a decrease in disability-adjusted life years (95% confidence interval, −2.92 to −0.78). An abbreviation key can be found online at http://www.jaad.org.

**Fig 2:**
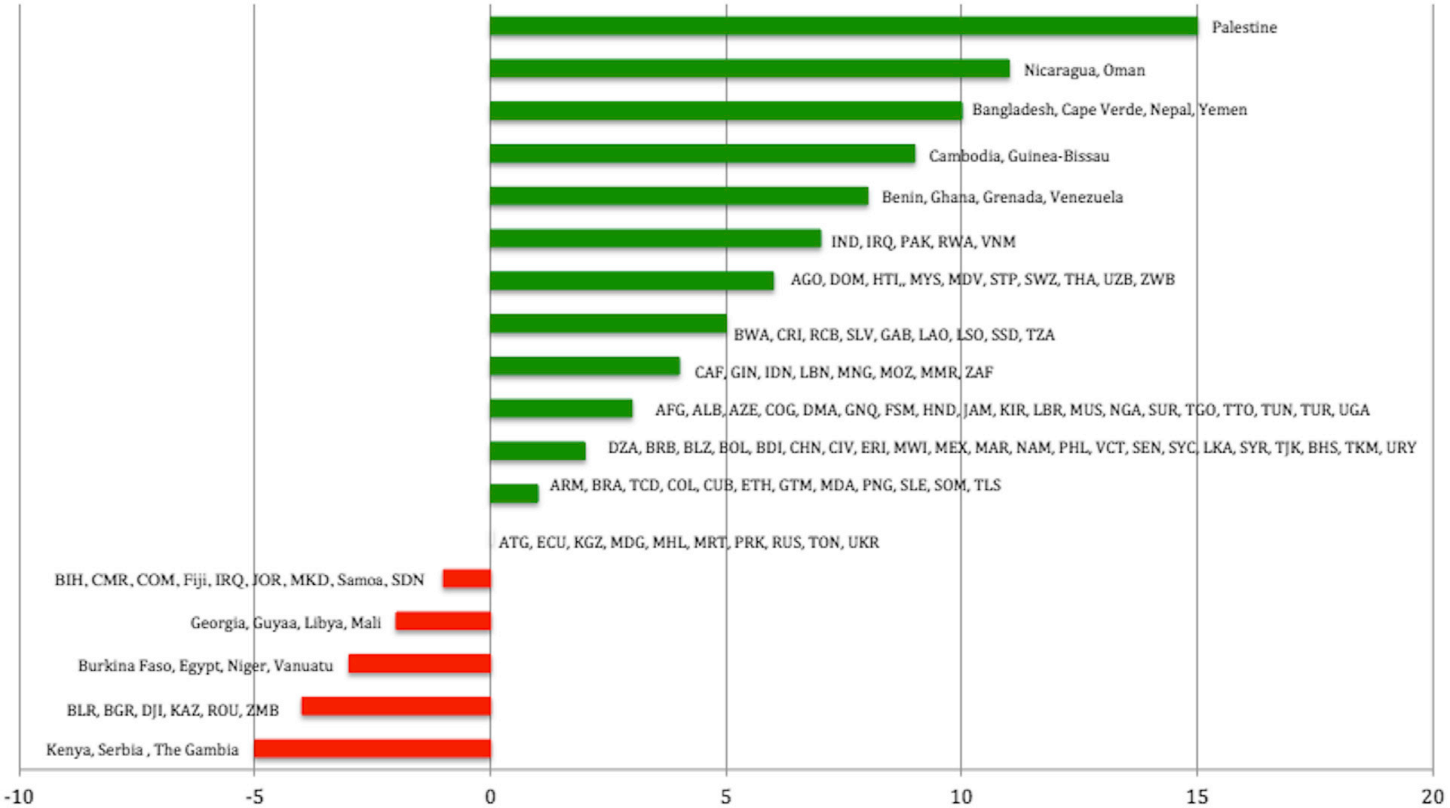
Percent change in disability-adjusted life years for developing countries. Negative values indicate a decrease in disability-adjusted life years (95% confidence interval, 2.04–3.29). An abbreviation key can be found online at http://www.jaad.org.

## Appendix Abbreviations used:

Poland – POL

Taiwan – TWN

Korea – KOR

Denmark – DNK

Finland – FIN

Greece – GRC

Italy – ITA

Luxembourg – LUX

Montenegro – MNE

The Netherlands – NLD

Slovakia – SVK

Australia – AUS

Cyprus – CYP

Japan – JPN

Latvia – LVA

Malta – MLT

Singapore – SGP

India – IND

Iran – IRN

Pakistan – PAK

Rwanda – RWA

Vietnam – VNM

Angola – AGO

Dominican Republic – DOM

Haiti – HTI

Malaysia – MYS

Maldives – MDV

Sao Tome and Principe – STP

Swaziland – SWZ

Thailand – THA

Uzbekistan – UZB

Zimbabwe – ZWB

Botswana – BWA

Costa Rica – CRI

Democratic Republic of the Congo – RCB

El Salvador – SLV

Gabon – GAB

Laos – LAO

Lesotho – LSO

South Sudan – SSD

Tanzania – TZA

Central African Republic – CAF

Guinea – GIN

Indonesia – IDN

Lebanon – LBN

Mongolia – MNG

Mozambique – MOZ

Myanmar – MMR

South Africa – ZAF

Afghanistan – AFG

Albania – ALB

Azerbaijan – AZE

Congo – COG

Dominica – DMA

Equatorial Guinea – GNQ

Federated States of Micronesia – FSM

Honduras – HND

Jamaica – JAM

Kiribati – KIR

Liberia – LBR

Mauritius – MUS

Nigeria – NGA

Suriname – SUR

Togo – TGO

Trinidad and Tobago – TTO

Tunisia – TUN

Turkey – TUR

Uganda – UGA

Algeria – DZA

Barbados – BRB

Belize – BLZ

Bolivia – BOL

Burundi – BDI

China – CHN

Cote d'Ivoire – CIV

Eritrea – ERI

Malawi – MWI

Mexico – MEX

Morocco – MAR

Namibia – NAM

Philippines – PHL

Saint Vincent and the Grenadines – VCT

Senegal – SEN

Seychelles – SYC

Sri Lanka – LKA

Syria – SYR

Tajikistan – TJK

The Bahamas – BHS

Turkmenistan – TKM

Uruguay – URY

Armenia – ARM

Brazil – BRA

Chad – TCD

Colombia – COL

Cuba – CUB

Ethiopia – ETH

Guatemala – GTM

Moldova – MDA

Panama – PA

Papua New Guinea – PNG

Sierra Leone – SLE

Somalia – SOM

Timor-Leste – TLS

Antigua and Barbuda – ATG

Ecuador – ECU

Kyrgyzstan – KGZ

Madagascar – MDG

Marshall Islands – MHL

Mauritania – MRT

North Korea – PRK

Russia – RUS

Tonga – TON

Ukraine – UKR

Bosnia and Herzegovina – BIH

Cameroon – CMR

Comoros – COM

Iraq – IRQ

Jordan – JOR

Macedonia – MKD

Sudan – SDN

Belarus – BLR

Bulgaria – BGR

Djibouti – DJI

Kazakhstan – KAZ

Romania – ROU

Zambia – ZMB
